# Synthesis and Structure–Activity Relationship of Thioacetamide-Triazoles against *Escherichia coli*

**DOI:** 10.3390/molecules27051518

**Published:** 2022-02-24

**Authors:** Suresh Dharuman, Miranda J. Wallace, Stephanie M. Reeve, Jürgen B. Bulitta, Richard E. Lee

**Affiliations:** 1 Department of Chemical Biology and Therapeutics, St. Jude Children’s Research Hospital, Memphis, TN 38105, USA; suresh.dharuman@stjude.org (S.D.); mjwallace@wustl.edu (M.J.W.); stephanie.reeve@stjude.org (S.M.R.); 2 Department of Pathology & Immunology, Division of Laboratory and Genomic Medicine, Washington University School of Medicine, St. Louis, MO 63110, USA; 3 Departments of Pharmaceutics and Pharmacotherapy and Translational Research, College of Pharmacy, University of Florida, Orlando, FL 31836, USA; jbulitta@cop.ufl.edu

**Keywords:** antimetabolite, 1,2,3-triazoles, gram-negative active compounds, antibiotics

## Abstract

Infections due to Gram-negative bacteria are increasingly dangerous due to the spread of multi-drug resistant strains, emphasizing the urgent need for new antibiotics with alternative modes of action. We have previously identified a novel class of antibacterial agents, thioacetamide-triazoles, using an antifolate targeted screen and determined their mode of action which is dependent on activation by cysteine synthase A. Herein, we report a detailed examination of the anti-*E. coli* structure–activity relationship of the thioacetamide-triazoles. Analogs of the initial hit compounds were synthesized to study the contribution of the aryl, thioacetamide, and triazole sections. A clear structure–activity relationship was observed generating compounds with excellent inhibition values. Substitutions to the aryl ring were generally best tolerated, including the introduction of thiazole and pyridine heteroaryl systems. Substitutions to the central thioacetamide linker section were more nuanced; the introduction of a methyl branch to the thioacetamide linker substantially decreased antibacterial activity, but the isomeric propionamide and *N*-benzamide systems retained activity. Changes to the triazole portion of the molecule dramatically decreased the antibacterial activity, further indicating that 1,2,3-triazole is critical for potency. From these studies, we have identified new lead compounds with desirable in-vitro ADME properties and in-vivo pharmacokinetic properties.

## 1. Introduction

**Figure 1:**
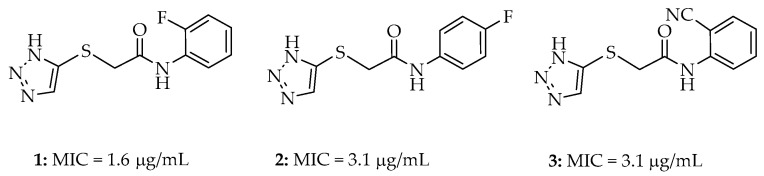
Hits from the screened library and MIC against *E. coli* K12.

The usage of antibiotics to treat and prevent bacterial infections has saved millions of people since their introduction. However, the overuse of antibiotics and the bacteria’s natural ability to develop resistance has led to a loss of effectiveness [1]. Antimicrobial resistance significantly increases the morbidity and mortality associated with bacterial infections in humans [2]. One reason for such a high mortality rate is the lack of alternative antibiotics to treat drug-resistant pathogens [3]. Therefore, there is a growing concern over antibiotic resistance and a need for the discovery of new drugs with novel mechanisms of action to treat resistant pathogens [4]. Previously, we have identified a thioacetamide-triazole hit series via a metabolically biased high-throughput screen against *E. coli* K12 in a search for novel antifolates (Figure 1) [5]. We demonstrated that these compounds function as prodrugs and are activated by the cysteine synthase A (CysK) enzyme. CysK plays a fundamental role in several key metabolic processes including cysteine biosynthesis and sulfur assimilation, and is also linked to the folate biosynthetic pathway through the downstream product homocysteine [5,6]. Thioacetamide triazoles (TATs) form a false product with the CysK substrate *O*-acetyl-L-serine [7] and thus inhibit the growth of *E. coli*. In continuation of our interest in identifying novel compounds for Gram-negative bacteria, in this study we explore the structure–activity relationships of the TAT series with the goal to develop potent lead compounds with excellent pharmacological properties.

## 2. Results

### 2.1. Synthesis

**Figure 2:**
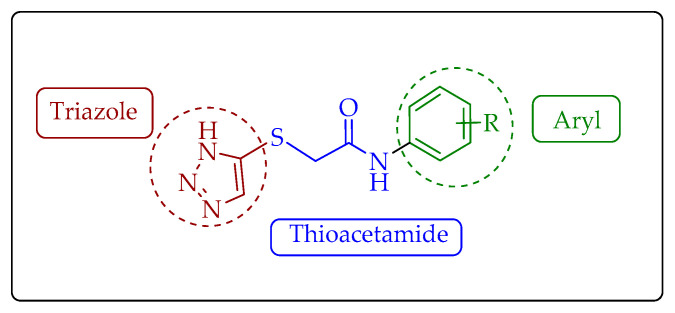
Aryl, thioacetamide and triazole sections of the initial lead examined in this SAR study.

To expand the structure–activity relationship (SAR) of the TAT series, we proposed a stepwise study to evaluate the contributions of the functional motifs found within the TAT scaffold. These series also allowed for the improvement of the physiochemical and pharmacological properties of our initial hits as we navigated our design between the competing requirements of CysK activation, Gram-negative intracellular accumulation, and pharmacokinetic exposure. Therefore, we focused on sequentially generating modifications to the aryl (right hand side), thioacetamide (middle) and triazole (left hand side) sections of the initial leads (Figure 2). 

**Scheme 1:**
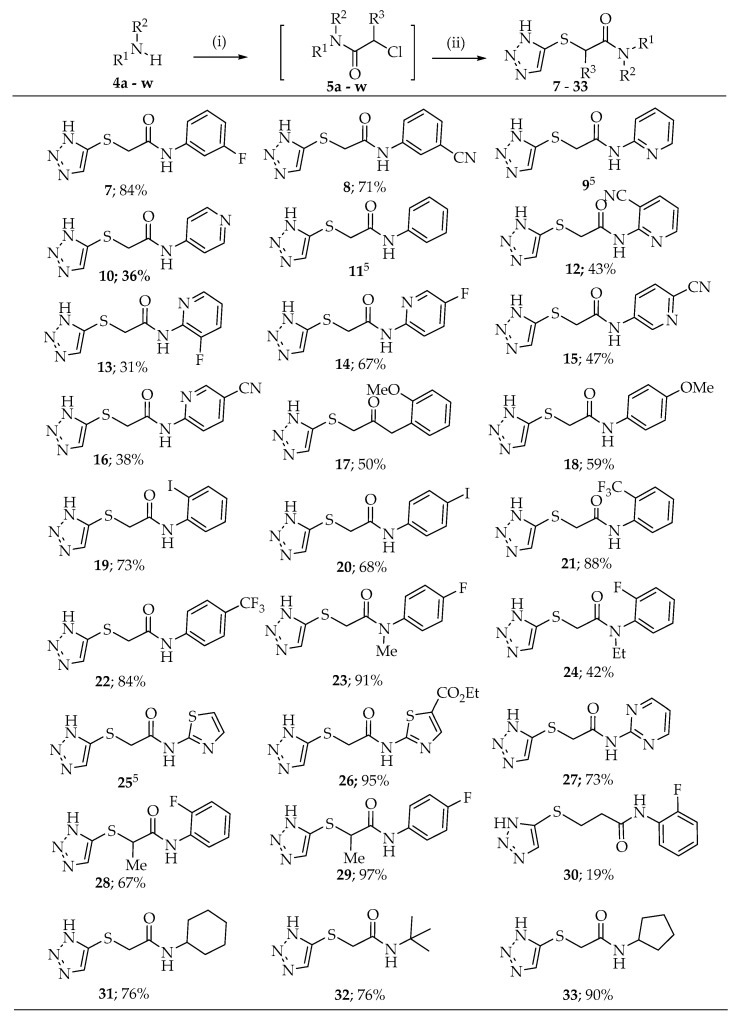
Reagents and conditions: (i) Chloroacetyl chloride or 2–chloropropionyl chloride, Et_3_N, CH_2_Cl_2_; 0 °C (ii) Sodium 1*H*-1,2,3-triazole-5-thiolate **6**, THF, 70 °C.

First, analogs were synthesized to probe the aryl region by incorporating various electron-donating, electron-withdrawing and sterically bulky groups on the phenyl aryl group (Scheme 1). The synthesis commenced from suitably substituted amines **4a**–**w**, which were converted to chloroacetamide derivatives **5a**–**w** [8]. Finally, the compounds were treated with sodium 1*H*-1,2,3-triazole-5-thiolate **6** (Scheme 1) to afford target TAT analogs **7**–**22** and **31**–**33**. In addition, *N*-methyl amide **23** and *N*-ethyl amide **24** derivatives were synthesized from *N*-methyl 4-fluoro aniline and *N*-ethyl-2-fluoroaniline via a similar scheme of coupling with chloroacetyl chloride and nucleophilic displacement with **6**. Using a similar approach, analogs with an alteration to the thioacetamide linker were generated using 2-chloropropionyl chloride and 3-chloropropionyl chloride, to give corresponding methyl branched derivatives **28** and **29** and the propionamide derivative **30**. 

**Scheme 2:**
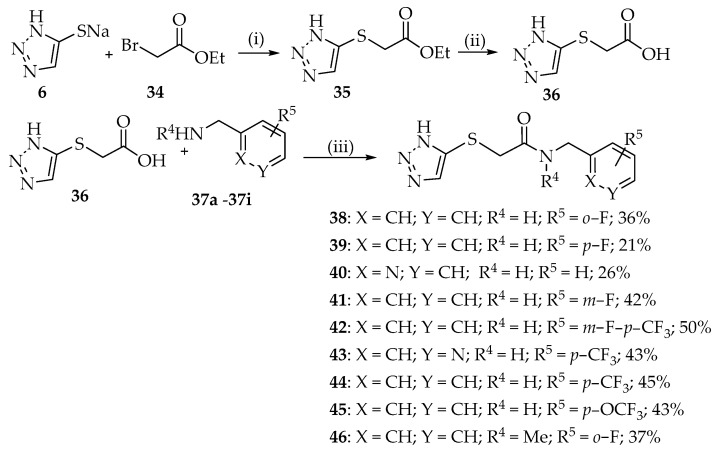
Reagents and conditions: (i) THF, 70 °C, 92% (ii) KOH, CH_3_OH, r.t, 98% (iii) EDC, HOBt, THF, DIPEA, r.t.

Next, we planned to incorporate a methylene group between the aryl and amide groups (Scheme 2). For this synthesis, the left hand side and linker section **36** was first assembled by reacting ethyl bromoacetate with **6** to produce compound **35**. Compound **35** was converted to free acid **36** with base hydrolysis [9]. Then, the acid was coupled with various benzyl amines using EDC, HOBt coupling to give compounds **38**–**46**, allowing for the facile introduction of structural diversity in the last step. 

**Scheme 3:**
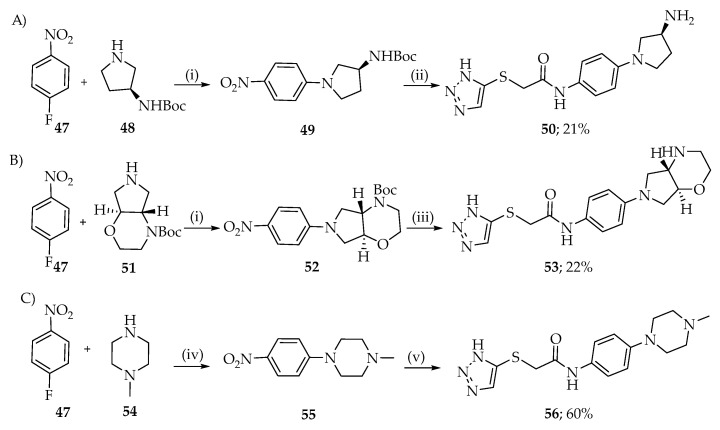
Reagents and conditions: (i) DIPEA, CH_3_CN, 90 °C (ii) (**A**) Raney Ni, H_2_, r.t, chloroacetyl chloride, 0 °C; (**B**) 1,2,3-Triazole-5-thiolate **6**, THF, 70 °C; (**C**) 4 M HCl in dioxane, r.t (iii) (**A**) Pd/C, H_2_, MeOH, chloroacetyl chloride, 0 °C; (**b**) 1, 2,3-Triazole-5-thiolate **6**, THF, 70 °C; (**C**) 4 M HCl in dioxane, r.t (iv) K_2_CO_3_, DMSO, 90 °C (v) (**a**) Pd/C, H_2_, MeOH, chloroacetyl chloride, 0 °C; (**B**) 1,2,3-Triazole-5-thiolate **6**, THF, 70 °C.

For the introduction of further right hand side aryl substitutions, we sought inspiration from the SAR of other antibiotic families. In fluoroquinolones, C-7 cycloalkyl amine substitutions are used to improve the antibacterial spectrum, solubility, and pharmacokinetic profiles of the antibiotic class, all properties we hoped to improve in the TAT series [10,11]. The C-7 fluoroquinolone substitution motifs are most commonly comprised of 5- or 6-membered cycloalkyl amines that contain an additional basic functional group, and consequently, we wanted to see if analogous functionalization could be beneficial to our compound series [12]. To test this hypothesis, we derivatized our initial hits with the 3-amino pyrrolidine, pyrrolo-oxazinyl, and *N*-methyl piperazine moieties. Compounds **50**, **53** and **56** were synthesized from 4-fluoro-nitrobenzene, as shown in Scheme 3. The nucleophilic displacement reaction of 4-fluoronitrobenzene **47** with boc-protected 3-aminopyrrolidine **48** in the presence of triethylamine yielded compound **49** [13]. Then, the nitro derivative **49** was reduced using Raney Ni to give amine, then treated with chloroacetyl chloride to provide the chloroacetamide derivative. Finally, the treatment of the chloroacetamide with thiolate **6** produced the final compound **50**. Similarly, compounds **53** and **56** were synthesized from amino derivatives **51** and **54**, respectively [14]. 

**Scheme 4:**
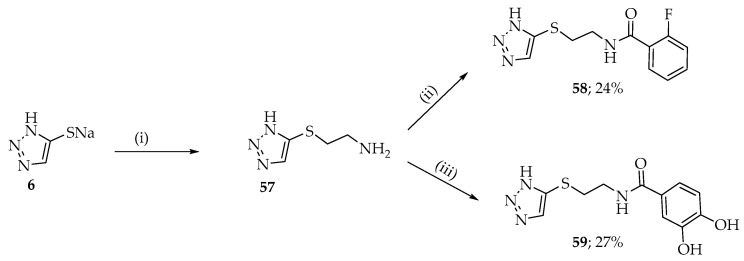
Reagents and conditions: (i) (**a**) *tert*-Butyl (2-bromoethyl)carbamate, THF, 80 °C; (**b**) 4 M HCl in dioxane, r.t (ii) EDC, HOBt, 2-fluorobenzoic acid, r.t (iii) EDC, HOBt, 3,4-dihydroxybenzoic acid, r.t.

To probe the importance of the central TAT phenylamide group, compounds **58** and **59**, in which the amide group was inverted, were synthesized. To achieve this, the thiolate **6** was treated with *tert*-butyl (2-bromoethyl)carbamate in THF to give the protected thiotriazole ethylamine, which was converted to the free amine **57** with acid. Compound **57** was then coupled with 2-fluorobenzoic acid to give **58**, and using a similar coupling protocol, **59** was synthesized from 3,4-dihydroxybenzoic acid (Scheme 4). 

**Scheme 5:**
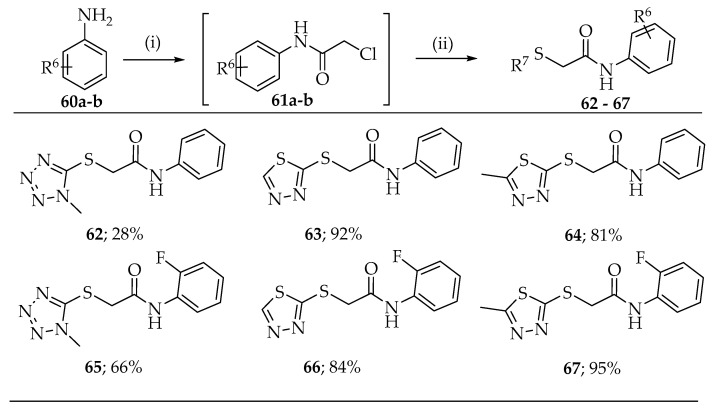
Reagents and conditions: (i) Chloroacetyl chloride, Et_3_N, CH_2_Cl_2,_ 0 °C; (ii) R^7^SH, Et_3_N, THF, 70 °C.

Finally, we prepared a series of compounds bearing various triazole analogs to explore structure–activity relationships on the triazole motif of the original hits. Compounds **62**–**67** were synthesized using a similar protocol as used in Scheme 1 (Scheme 5). Amines **60a**–**b** were converted to chloroacetyl derivatives **61a**–**b** and coupled with respective thiazoles to give compounds **62**–**67**.

**Scheme 6:**
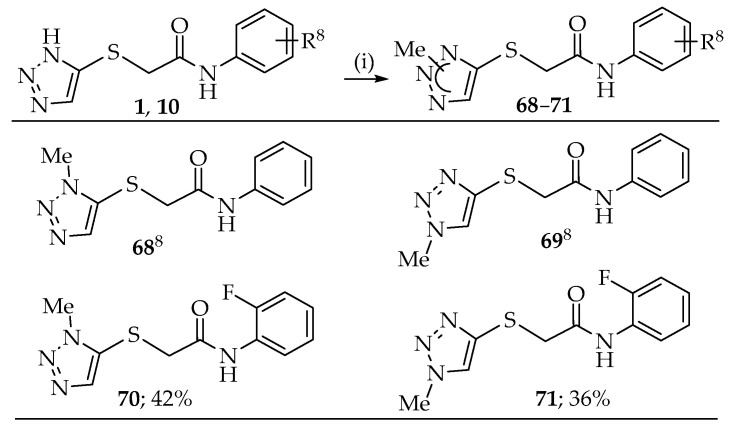
Reagents and conditions: (i) MeI, Cs_2_CO_3_, CH_3_CN, r.t.

To examine the contribution of the triazole N-H, the corresponding N-Me derivatives **68** and **69** were prepared by the methylation of **10** using cesium carbonate and iodomethane. Similarly, compounds **70** and **71** were synthesized in good yields (Scheme 6). 

### 2.2. Antibacterial Studies

To explore the SAR around the right hand aryl portion of the TAT series (Table 1), compounds were evaluated for their in vitro antibacterial activity against E. coli in the M9-based minimal media ATCC 2511. Growth inhibition was also evaluated in methionine supplemented media, as it has been shown to rescue cellular inhibition of the initial hit compounds [5] as well as other antifolates [15,16]. An MIC shift between the two medias was used to indicate that antibacterial activity of the compounds remained on target, and this was happily seen with all of the most MIC active compounds generated in this study. Within the first series of compounds in Table 1, modifications to the phenyl ring are generally well tolerated. MICs remain within 2-fold of the initial hit **1** for the fluoro- and cyano- substituted compounds **1**–**8**. The installation of a 2-pyridyl nitrogen **9**, **13**, **14**, **16** was generally well tolerated, with the exception of the 4-pyridyl analog **10**, which showed an 8-fold loss in MIC activity. Incorporating the electron-donating group -OMe and sterically bulky iodine group in both *ortho* and *para* position, **17**–**20**, maintained activity, whereas the introduction of the strong electron-withdrawing -CF_3_ group, **21** and **22**, led to poorer MIC activity. Blocking the linker amide NH with the Me or Et (**23** and **24**) groups also reduced activity. The phenyl ring isosteres—thiazoles **25** and **26**—both retained good MIC activity. Adding a methyl branch to the thioacetamide linker **28** and **29** led to a striking loss of activity. Replacement of the aryl group with the aliphatic groups cyclohexyl **31**, cyclopentyl **32**, *tert*-butyl groups **33** all reduced activity.

Substituting the phenylaniline TAT motif with a benzylamine to remove the aniline motif was explored in a second series (**38**–**46**, Table 2). The simple fluorine benzamide substitutions **38**, **39**, **41** maintained good MIC activity with only a two-fold MIC shift from the values of the corresponding phenyl amides **1**, **3**, **7**. However, the other analogs, **40** and **42**–**46**, showed decreased antibacterial activity against *E. coli*. This series indicates that benzamides are slightly less preferable from an MIC activity standpoint, but do offer optional extra chemical diversity that could be utilized in future series.

The introduction of the fluoroquinolone C7 basic motif into the *para*-position of the right hand side phenyl ring in compounds **50**, **53**, and **56**, in an effort to improve the pharmacokinetic profile of the series, was not successful. None of these compounds show any notable anti-*E.coli* activity (Table 3). Compounds **58** and **59**, in which the amide group was inverted, were synthesized to determine if modification could be made to the central TAT phenylamide group. These compounds only had moderate activity. To assess whether or not the lack of antibacterial activity accompanying the modifications of the phenyl ring is due to poor cellular uptake, we examined the accumulation of **50**, **53**, **56**, **58**, **59** and **1** in an LC-MS/MS based assay (Table 3 and Table S3) [17]. This assay indicated that the lack of MIC activity of **50**, **53** and **56** was not due to a much lower drug accumulation within the cell than **1**, and that incompatibility with the biochemical target CysK is most likely responsible for poor antimicrobial activity. In terms of general accumulation, all these analogs tested were lower than the ciprofloxacin control, suggesting room for improvement. The catechol **59**, designed to capitalize on outer membrane siderophore iron-dependent transport mechanisms, showed the lowest accumulation of the whole series, suggesting outer membrane permeability may not be limiting for the series [18]. 

To complete our SAR analysis, a small series of compounds with various triazole analogs were evaluated to determine if any modifications to the left hand side could be tolerated. The *N*-methyl tetrazole **62**, thiadiazole **63**, and methyl-thiadiazole **64** analogs were inactive (Table 4). Similarly, compounds **65**–**71** were found to be inactive. These results are consistent with our prior mode of action studies that indicate the triazole NH is required for CysK activation [5]. 

To examine the potential for the further development of the newly synthesized analogs, in vitro ADME properties of promising compounds **9** and **25**, and our original hits **1**, **2**, and **3** were examined (Table 5 and Table S1). Both compounds **9** and **25** demonstrated improved solubility, and plasma and metabolic stability over compound **1**. Encouraged by the improved mouse plasma and microsomal stability, the mouse pharmacokinetic profile of **25** by intravenous dosing was determined. Compound **25** showed a moderate exposure (AUC_inf_ 2807 h·ng/mL) and a mean elimination half-life of 0.55 h (Table S2). 

## 3. Discussion

**Figure 3:**
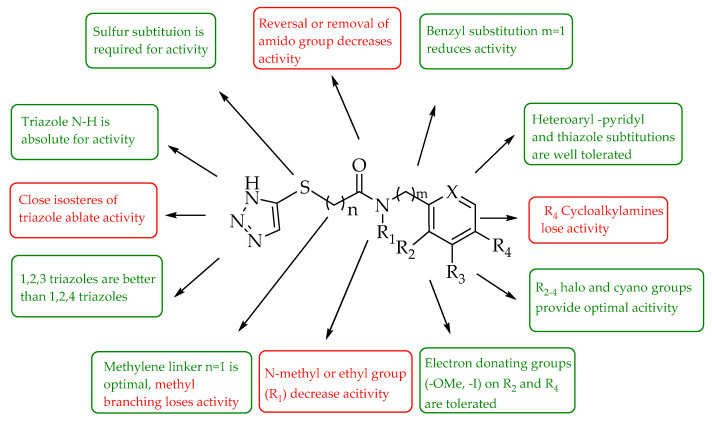
SAR analysis of thioacetamide compounds against *E. coli* K12.

In this study, the structure–activity relationship of the TAT series was closely examined by targeted synthesis and anti-*E.coli* testing. The results, joined with observations from our initial publication, [5] provide a full picture of this novel anti-Gram negative chemotype. The combined SAR is summarized in Figure 3, showing a clear structure–activity relationship with the tightest structural requirements for the thio-linked triazole ring. This is consistent with our prior finding that triazole NH is a critical acceptor for the CysK activation and consequential false product formation. Substitutions to the right-hand side aryl ring were generally best tolerated, including the introduction of thiazole and pyridine heteroaryl systems to increase the pharmacological properties. Substitutions to the central acetamide linker section were more nuanced, the introduction of a methyl branch substantially decreased antibacterial activity, but the isomeric propionamide and *N*-benzamide systems retained activity. 

A primary challenge in Gram-negative drug discovery is understanding the SAR rules of MIC activity, which is a combination of the ability of the inhibitor to penetrate and accumulate into the bacterial cytoplasm, and its ability to inhibit the molecular target [19]. In this case, the TAT series has many desirable properties previously reported for Gram-negative entry, including a low molecular weight and polarity range [20]. To further understand the SAR, we performed a retrospective analysis of the physiochemical and MIC properties of all the compounds generated in the series that had the optimal thioacetamide-triazole motifs required and diverging only in the aryl section substitution (Figure S1). Compounds **1**, **7**, **9**, and **25**, with the best MIC values (MIC ≤ 3.1 µg/mL), all clustered with LogD 0.54–2.25 and TPSA 70–110 Å^2^ ranges, consistent with the properties of other Gram-negative drugs that are less susceptible to efflux [21,22]. 

To complement this study a subseries of compounds was generated, designed to increase intracellular accumulation by the incorporation of basic cycloalkyl rings to the aryl motif. This strategy was not successful in increasing intracellular accumulation for the TAT series and was likely incompatible with the intracellular molecular target, resulting in a loss in MIC activity. In this series, smaller TAT analogs appear to be advantageous.

The range of TAT analogs generated allows for the selection of compounds with desirable pharmacological properties and the potential for future development. The plasma stability of our initial lead **1** was low, likely due to inactivation by mouse carboxyesterases, a known issue with similar amide systems [23]. Though plasma stability was less of an issue for human plasma, the development path required mouse efficacy experiments and better compounds were sought, this analysis led us to prioritize compound **25** for further development due to its excellent MIC, human and mouse plasma stabilities, and microsomal stabilities. Pharmacokinetic profiling of **25** suggested it may be suitable for further evaluation. 

## 4. Materials and Methods 

### 4.1. General Experimental Procedure

All solvents used for chromatography and liquid chromatography were purchased from Aldrich. Flash column chromatography silica cartridges were obtained from Biotage Inc (Biotage, LLC, Charlotte, NC, USA). Reactions were monitored by thin-layer chromatography (TLC) on pre-coated Merch 60 F254 silica gel plates and visualized using UV light (254 nm). A Biotage FLASH column chromatography system (Biotage, LLC, Charlotte, NC, USA) was used to purify mixtures. ^1^H NMR spectra were recorded on a Varian INOVA-500 spectrometer or on a Bruker 400 MHz NMR spectrometer (Bruker Scientific LLC, Billerica, MA, USA). Chemical shifts (δ) are reported in parts per million, relative to the residual solvent peak or internal standard (tetramethylsilane), and coupling constants (*J*) are reported in hertz (Hz). Purity of the products was confirmed by UPLC/MS (the Waters Acquity) (Waters Corporation, Milford, MA, USA). Optical rotations were analyzed on a Jasco P-1010 polarimeter instrument with a path length of 1 dm (589 nm) and reported as follows: [α]_D_^T^ (c in grams per 100 mL of solvent). Melting points were recorded using Büchi melting point B-545 instrument (Büchi Corporation, New Castle, DE, USA). Analytical data are given for active compounds, and data for all other compounds are provided in the Supplementary Information. 

2-[(1*H*-1,2,3-triazol-4-yl)sulfanyl]-*N*-(3-fluorophenyl)acetamide (**7**): To a stirred solution of substituted amine (500 mg, 4.55 mmoL) in CH_2_Cl_2_ (7.0 mL) was added triethylamine (0.63 mL, 4.55 mmoL) and chloroacetyl chloride (0.35 mL, 4.55 mmoL) at 0 °C. After stirring the reaction mixture for 1 h, diluted with CH_2_Cl_2_ (10 mL), washed with NaHCO_3_, the organic layer was dried over Na_2_SO_4_ and concentrated under high vacuum to give crude chloroacetyl derivative. To a crude chloroacetyl derivative in THF (5.0 mL) was added sodium 1*H*-1,2,3-triazole-5-thiolate (400 mg, 2.13 mmoL) and heated at 70 °C for 1 h. The solids were filtered off, and the filtrate was evaporated under a high vacuum to give a crude product. The crude product was purified over silica-gel column chromatography (Eluents: 20–50% EtOAc in hexane) to produce final product **7** (450 mg, 84%) as a white solid. m.p. 136–136 °C; ^1^H NMR (500 MHz, CD_3_OD) *δ* 7.86 (s, 1H), 7.48 (d, *J* = 11.3 Hz, 1H), 7.28 (t, *J* = 7.3 Hz, 1H), 7.20 (d, *J* = 8.2 Hz, 1H), 6.82 (t, *J* = 8.5 Hz, 1H), 3.71 (s, 2H); ^13^C NMR (125 MHz, CD_3_OD) *δ* 168.2, 162.9 (d, *J* = 243.0 Hz), 142.2–138.7 (m), 129.8 (d, *J* = 9.4 Hz), 110.3 (dd, *J* = 21.3, 2.3 Hz), 106.7 (dd, *J* = 26.5, 2.8 Hz), 38.7; HRMS (ESI) m/z calcd for C_10_H_10_FN_4_OS [M + H]^+^, 253.0559; found, 253.0557.

2-[(1*H*-1,2,3-triazol-4-yl)sulfanyl]-*N*-(3-cyanophenyl)acetamide (**8**): Compound **8** (380 mg, 71%) was analogously synthesized as **7**, as a yellow solid. m.p. 158–159 °C; ^1^H NMR (500 MHz, CD_3_OD) *δ* 8.00 (t, *J* = 1.8 Hz, 1H), 7.88 (s, 1H), 7.73 (dt, *J* = 8.1, 1.7 Hz, 1H), 7.57–7.24 (m, 2H), 3.73 (s, 2H); ^13^C NMR (125 MHz, CD_3_OD) *δ* 38.7, 112.4, 118.1, 122.5, 123.9, 127.3, 129.7, 139.3, 168.4; HRMS (ESI) m/z calcd for C_11_H_10_N_5_OS [M + H]^+^, 260.0606; found, 260.0598.

2-[(1*H*-1,2,3-triazol-4-yl)sulfanyl]-*N*-(3-fluoropyridin-2-yl)acetamide (**13**): Compound **13** (50 mg, 31%) was analogously synthesized as **7**, as a white solid. m.p. 99–100 °C; ^1^H NMR (500 MHz, CD_3_OD) *δ* 8.20 (d, *J* = 4.8 Hz, 1H), 7.90 (s, 1H), 7.64 (dd, *J* = 10.2, 8.0 Hz, 1H), 7.29 (dt, *J* = 8.4, 4.2 Hz, 1H), 3.85 (s, 2H); ^13^C NMR (125 MHz, CD_3_OD) *δ* 168.8, 151.9 (d, *J* = 259.5 Hz), 143.4 (d, *J* = 5.5 Hz), 139.5 (d, *J* = 13.1 Hz), 131.2, 127.0–118.7 (m), 38.1; HRMS (ESI) m/z calcd for C_9_H_9_FN_5_OS [M + H]^+^, 254.0512; found, 254.0501.

2-[(1*H*-1,2,3-triazol-4-yl)sulfanyl]-*N*-(5-fluoropyridin-2-yl)acetamide (**14**): Compound **14** (180 mg, 67%) was analogously synthesized as **7**, as a white solid. m.p. 151–153 °C; ^1^H NMR (500 MHz, CD_3_OD) *δ* 8.18 (d, *J* = 3.1 Hz, 1H), 8.11 (dd, *J* = 9.2, 4.1 Hz, 1H), 7.86 (s, 1H), 7.57 (ddd, *J* = 9.2, 8.0, 3.1 Hz, 1H), 3.78 (s, 2H); ^13^C NMR (125 MHz, CD_3_OD) *δ* 168.19, 156.55 (d, *J* = 249.9 Hz), 147.84 (d, *J* = 2.3 Hz), 135.09 (d, *J* = 25.6 Hz), 124.71 (d, *J* = 19.8 Hz), 114.91 (d, *J* = 4.5 Hz), 38.41; HRMS (ESI) m/z calcd for C_9_H_9_FN_5_OS [M + H]^+^, 254.0512; found, 254.0507.

2-[(1*H*-1,2,3-triazol-4-yl)sulfanyl]-*N*-(6-cyanopyridin-3-yl)acetamide (**15**): Compound **15** (250 mg, 47%) was analogously synthesized as **7**, as a white solid. m.p. 198–199 °C; ^1^H NMR (500 MHz, DMSO-*d*_6_) *δ* 10.89 (s, 1H), 8.83 (d, *J* = 2.5 Hz, 1H), 8.23 (dd, *J* = 8.6, 2.5 Hz, 1H), 7.99 (d, *J* = 8.6 Hz, 1H), 3.88 (s, 2H); ^13^C NMR (125 MHz, DMSO-*d*_6_) *δ* 38.8, 118.1, 126.3, 126.6, 130.2, 139.2, 142.3, 168.5; HRMS (ESI) m/z calcd for C_10_H_9_N_6_OS [M + H]^+^, 261.0559; found, 261.0554.

2-[(1*H*-1,2,3-triazol-4-yl)sulfanyl]-*N*-(5-cyanopyridin-2-yl)acetamide (**16**): Compound **16** (25 mg, 38%) was analogously synthesized as **7**, as a brown solid. m.p. 118–119 °C; ^1^H NMR (500 MHz, CD_3_OD) *δ* 8.63 (d, *J* = 2.4 Hz, 1H), 8.25 (d, *J* = 8.7 Hz, 1H), 8.08 (dd, *J* = 8.7, 2.3 Hz, 1H), 7.38 (s, 1H), 3.82 (s, 2H); ^13^C NMR (125 MHz, CD_3_OD) *δ* 38.6, 104.6, 113.2, 116.5, 141.4, 151.7, 154.3, 168.9; HRMS (ESI) m/z calcd for C_10_H_9_N_6_OS [M + H]^+^, 261.0559; found, 261.0557.

2-[(1*H*-1,2,3-triazol-4-yl)sulfanyl]-*N*-(2-methoxyphenyl)acetamide (**17**): Compound **17** (300 mg, 50%) was analogously synthesized as **7**, as a white solid. m.p. 99–100 °C; ^1^H NMR (500 MHz, CD_3_OD) *δ* 7.98 (dd, *J* = 8.0, 1.7 Hz, 1H), 7.85 (s, 1H), 7.08 (ddd, *J* = 8.2, 7.5, 1.6 Hz, 1H), 6.98 (dd, *J* = 8.2, 1.3 Hz, 1H), 6.89 (td, *J* = 7.7, 1.3 Hz, 1H), 3.85 (s, 3H), 3.80 (s, 2H); ^13^C NMR (125 MHz, CD_3_OD) *δ* 38.3, 110.4, 120.1, 121.1, 124.8, 126.6, 149.7, 167.9; HRMS (ESI) m/z calcd for C_11_H_13_N_4_O_2_S [M + H]^+^, 265.0759; found, 265.0760.

2-[(1*H*-1,2,3-triazol-4-yl)sulfanyl]-*N*-(4-methoxyphenyl)acetamide (**18**): Compound **18** (350 mg, 59%) was analogously synthesized as **7**, as a white solid. m.p. 130–131 °C; ^1^H NMR (500 MHz, CD_3_OD) *δ* 7.84 (s, 1H), 7.68–7.29 (m, 2H), 7.17–6.29 (m, 2H), 3.77 (s, 3H), 3.68 (s, 2H); ^13^C NMR (125 MHz, CD_3_OD D) *δ* 38.7, 54.4, 113.6, 121.7, 131.0, 131.4, 137.7, 156.7, 167.8; HRMS (ESI) m/z calcd for C_11_H_13_N_4_O_2_S [M + H]^+^, 265.0759; found, 265.0760.

2-[(1*H*-1,2,3-triazol-4-yl)sulfanyl]-*N*-(2-iodophenyl)acetamide (**19**): Compound **19** (310 mg, 73%) was analogously synthesized as **7**, as a brown solid. m.p. 122–124 °C; ^1^H NMR (500 MHz, CD_3_OD) *δ* 7.89 (s, 1H), 7.87 (d, *J* = 8.1 Hz, 1H), 7.62 (d, *J* = 8.1 Hz, 1H), 7.43–7.21 (m, 1H), 6.96 (td, *J* = 7.6, 1.7 Hz, 1H), 3.83 (s, 2H); ^13^C NMR (125 MHz, CD_3_OD) *δ* 38.0, 93.1, 125.5, 127.4, 128.6, 131.0, 139.1, 168.4; HRMS (ESI) m/z calcd for C_10_H_10_IN_4_OS [M + H]^+^, 360.9620; found, 360.9622.

2-[(1*H*-1,2,3-triazol-4-yl)sulfanyl]-*N*-(4-iodophenyl)acetamide (**20**): Compound **20** (250 mg, 68%) was analogously synthesized as **7**, as a brown solid. m.p. 166–168 °C; ^1^H NMR (500 MHz, CD_3_OD) *δ* 7.83 (s, 1H), 7.74–7.54 (m, 2H), 7.34 (d, *J* = 8.7 Hz, 2H), 3.69 (s, 2H); ^13^C NMR (125 MHz, CD_3_OD) *δ* 38.8, 86.7, 121.6, 131.3, 137.5, 138.1, 168.1; HRMS (ESI) m/z calcd for C_10_H_10_IN_4_OS [M + H]^+^, 360.9620; found, 360.9615.

Ethyl 2-{2-[(1*H*-1,2,3-triazol-4-yl)sulfanyl]acetamido}thiazole-5-carboxylate (**26**): Compound **26** (60 mg, 95%) was analogously synthesized as **7**, as a yellow solid. m.p. 172–174 °C; ^1^H NMR (500 MHz, DMSO-*d*_6_) δ 8.14 (s, 1H), 7.95 (s, 1H), 4.27 (q, *J* = 7.1 Hz, 2H), 3.92 (s, 2H), 1.28 (t, *J* = 7.1 Hz, 3H); ^13^C NMR (125 MHz, DMSO-*d*_6_) *δ* 14.6, 38.8, 61.4, 121.9, 145.6, 161.8, 162.4, 168.3; HRMS (ESI) m/z calcd for C_10_H_12_N_5_O_3_S_2_ [M + H]^+^, 314.0382; found, 314.0378.

3-[(1*H*-1,2,3-triazol-4-yl)sulfanyl]-*N*-(2-fluorophenyl)propenamide (**30**): Compound **30** (10 mg, 19%) was analogously synthesized as **7** using 3-chloropropanoyl chloride as a reagent, as a white solid. m.p. 100–102 °C; ^1^H NMR (500 MHz, CD_3_OD) *δ* 7.86 (d, *J* = 8.1 Hz, 2H), 7.30–7.04 (m, 3H), 3.22 (t, *J* = 7.0 Hz, 2H), 2.78 (t, *J* = 7.1 Hz, 2H); ^13^C NMR (125 MHz, CD_3_OD) *δ* 170.9, 154.3 (d, *J* = 245.5 Hz), 135.6, 125.6 (d, *J* = 7.5 Hz), 124.1 (d, *J* = 61.3 Hz), 115.0 (d, *J* = 19.7 Hz), 36.2, 29.9; HRMS (ESI) m/z calcd for C_11_H_12_FN_4_OS [M + H]^+^, 267.0716; found, 267.0710.

2-[(1*H*-1,2,3-triazol-5-yl)sulfanyl]-*N*-(2-fluorobenzyl)acetamide (**38**): To a solution of acid **36 [9]** (100 mg, 0.63 mmoL), EDC (98 mg, 0.63 mmoL), HOBt (96 mg, 0.63 mmoL) and 2-fluoro-benzylamine (86 μL, 0.75 mmoL) in THF (2 mL) was added *N*,*N*-diisopropylethylamine (109 μL, 0.63 mmoL) at room temperature. The reaction mixture was stirred for 15min before it was evaporated under high vacuum to give crude product. The crude product was purified on reverse phase silica-gel column chromatography (Eluents: 0–3% acetonitrile in water) to give pure product **38** (60 mg, 36%), as a white solid. m.p. 102–103 °C; ^1^H NMR (500 MHz, CD_3_OD) *δ* 7.96 (s, 1H), 7.36–7.18 (m, 2H), 7.16–7.00 (m, 2H), 4.40 (s, 2H), 3.66 (s, 2H); ^13^C NMR (125 MHz, CD_3_OD) *δ* 169.5, 160.725 (d, *J* = 245.3 Hz), 137.5, 130.7, 130.1–128.4 (m), 124.8 (d, *J* = 14.9 Hz), 123.9 (d, *J* = 3.6 Hz), 114.8 (d, *J* = 21.4 Hz), 37.4, 36.9 (d, *J* = 4.7 Hz); HRMS (ESI) m/z calcd for C_11_H_12_FN_4_OS [M + H]^+^, 267.0716; found, 267.0708.

2-[(1*H*-1,2,3-triazol-5-yl)sulfanyl]-*N*-(4-fluorobenzyl)acetamide (**39**): The compound **39** (35 mg, 21%) was synthesized analogously as compound **38** from 4-fluoro-benzyl amine, as a white solid. m.p. 122–124 °C; ^1^H NMR (500 MHz, CD_3_CN) *δ* 7.71 (s, 1H), 7.40 (s, 1H), 7.26–7.19 (m, 2H), 7.14–6.95 (m, 2H), 4.33 (d, *J* = 6.1 Hz, 2H), 3.62 (s, 2H); ^13^C NMR (125 MHz, CD_3_CN) *δ* 168.8, 162.4 (d, *J* = 242.5 Hz), 135.7 (d, *J* = 3.1 Hz), 131.8, 129.7 (d, *J* = 8.2 Hz), 115.5 (d, *J* = 21.6 Hz); HRMS (ESI) m/z calcd for C_11_H_12_FN_4_OS [M + H]^+^, 267.0716; found, 267.0713.

### 4.2. Minimal Inhibitory Concentration Testing

MICs were determined following CLSI broth microdilution susceptibility M100 guidelines [24]. Compound solutions were prepared in DMSO and serially diluted 1:2 in 100 μL/well in 96 well round-bottom plates. *E. coli* K12 MG1655 was acquired from ATCC and strictly maintained in the M9-based media ATCC 2511. ATCC 2511 was also used for all further cultivation and MIC testing. Bacteria were grown from a single colony to a mid-log OD_600_ and frozen in 0.5 mL aliquots. These aliquots were used to directly inoculate the assay plates by diluting to an OD_600_ of 0.001 and plating 100 μL per well, allowing for ~10^5^ CFU to be added to each well. After 16 h of incubation at 37 °C, the MIC was determined at the concentration of compound at which no visible growth is detected. 

### 4.3. Whole Cell Accumulation in E. coli K12 

Drug uptake in whole cell *E. coli* K12, was performed following a modified protocol adapted from previously published studies [17,25,26,27]. *E. coli* was grown in LB media until a mid-log phase (OD_600_ of 0.7–0.8). Cells were harvested (7500× *g*, 15 min), washed twice with 40 mL of PBS and resuspended to a final volume of 3.5mL per 100 mL of initial culture. One milliliter of concentrated cell suspension was incubated with antibiotic at a final concentration of 100 mM of antibiotic for 10 min under shaking conditions at 37 °C. After incubation, 800 mL of antibiotic-exposed cells were spun (3 min, 13,000× *g*) through 700 mL of a 9:1 mix of AR20 and high temperature silicon oils (cooled to −80°C). The supernatant of silicone oil and free compound in PBS was carefully removed from pellet. Intracellular concentration of antibiotics within the bacterial cells was then determined. 

For lysis, cells were resuspended in 200 mL of water and lysed by three freeze-thaw cycles of 3 min each in liquid nitrogen and in a 60 °C water bath. Lysed cells were pelleted and 150 mL of aqueous extract was transferred to a clean 1.5 mL microcentrifuge tube. Cell debris was resuspended in the remaining 50 mL of water and then extracted with 100 mL of methanol. Cells were again centrifuged, 100 mL of the methanol extract were removed and combined with the water supernatant. The extracts were allowed to rest at room temperature for 1 h before being centrifuged for 10 min at 20,000× *g*. Finally, extracts were filtered through a 0.22mm spin column to remove any remaining cellular debris. 

Samples were analyzed with a Waters Acquity M Class series UPLC system and Xevo G2 QTOF tandem MS/MS with Zspray. Using a Phenomenex Kinetex 2.6 μm XB-C18, 100 Å (300 μm × 150 mm) column with solvent A, 0.1% formic acid in water, and solvent B, 0.1% formic acid in acetonitrile, 250 nL of extract were separated. The inlet method for these samples utilized a flow rate of 8 μL min^−1^ with the following gradient: 0−4 min, 99.9% solvent A and 0.1% solvent B; 4–5 min, 10% solvent A and 90% solvent B; 5–6 min, 99.9% solvent A and 0.1% solvent B. Tandem mass spectra were acquired using conditions presented in Table S3. High-resolution spectra were calibrated by co-infusion of 2 ng/mL leucine enkephalin lockspray (Waters). Data were quantified using Waters MassLynx software where the AUC was determined by integrating the daughter peak of the parent ion. Concentrations of the unknown compounds were determined by the linear fit of the standard curve. Concentrations were normalized to the culture density, determined via titers, to 10^10^ CFU and are reported as the average of three biological replicates. 

### 4.4. In Vitro ADME Profiling

The in vitro ADME values (solubility, mouse plasma stability, human plasma stability, mouse metabolic stability, and human metabolic stability) were determined as previously reported [28,29].

### 4.5. Pharmacokinetic Evaluation in Mice

Pharmacokinetic parameters of **25** in plasma following a single intravenous dose of 10mg/kg administration to male Balb/c mice were done at SAI LifeSciences Ltd., Telangana, India (Table S2). Briefly, nine male mice were used in this study. All the animals were administered with **25** solution formulation in 5% NMP, 5% Solutol HS-15 and 90% Normal saline intravenously at 10 mg/kg dose. Blood samples (~60 μL) were collected from retro orbital plexus under light isoflurane anesthesia at following time points at Pre-dose, 0.08, 0.25, 0.5, 1, 2, 4, 8 and 24 h. Blood samples were collected from a set of three mice at each time point (sparse sampling) in labeled microcentrifuge tube containing 20% K_2_EDTA solution as anticoagulant. Plasma samples were separated by centrifugation of whole blood and stored below −70 ± 10 °C until bioanalysis. All samples were processed for analysis by protein precipitation using acetonitrile (ACN) and analyzed with fit-for-purpose LC/MS/MS method (LLOQ: 5.04 ng/mL). Pharmacokinetic parameters were calculated using the non-compartmental analysis tool of Phoenix WinNonlin (Version 7.0). All procedures of the study were in accordance with the guidelines provided by the Committee for the Purpose of Control and Supervision of Experiments on Animals (CPCSEA) as published in The Gazette of India, 15 December 1998. Prior approval of the Institutional Animal Ethics Committee (IAEC) was obtained before initiation of the study.

## Figures and Tables

**Table 1:** MIC (μg/mL) of aryl analogs **1**–**30** against *E. coli* K12.

Compound	Structure	*E. coli* K12 MIC_90_ (μg/mL) *^a^*		Compound	Structure	*E. coli* K12 MIC_90_ (μg/mL) *^a^*	
		N.S.	+20 μg/mL Methionine			N.S.	+20 μg/mL Methionine
**1**	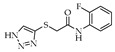	1.6	12.5	**19**	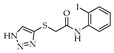	3.1	n.d.
**2**	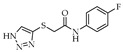	3.1	12.5	**20**	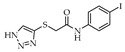	6.3	25
**3**	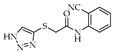	3.1	12.5	**21**	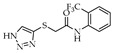	25.0	n.d.
**7**	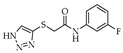	1.6	12.5	**22**	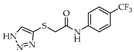	25	n.d.
**8**	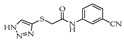	3.1	12.5	**23**	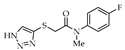	25	n.d.
**9**	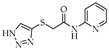	1.6	12.5	**24**	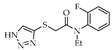	12.5	25
**10**	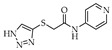	12.5	50	**25**	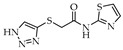	3.1	12.5
**11**	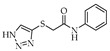	1.6	6.3	**26**	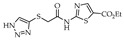	3.1	12.5
**12**	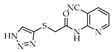	25.0	n.d	**27**	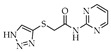	25	n.d.
**13**	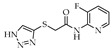	6.3	25	**28**	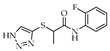	>200	n.d.
**14**	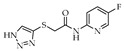	3.1	12.5	**29**	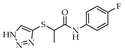	100	n.d.
**15**	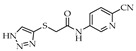	6.3	50	**30**	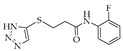	6.3	25
**16**	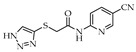	3.1	6.3	**31**	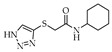	25	n.d.
**17**	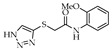	6.3	n.d.	**32**	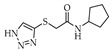	12.5	25
**18**	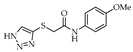	3.1	12.5	**33**	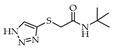	12.5	25.0

*^a^* = Chloramphenicol = 1.6 μg/mL, Sulfamethoxazole = 0.98 μg/mL, and Trimethoprim = 0.98 μg/mL were used as controls; N.S. = No Supplement; n.d. = Not determined.

**Table 2:** MIC (μg/mL) of benzamide analogs **38**–**46** against *E. coli* K12.

Item	Structure	*E. coli* K12 MIC_90_ (μg/mL)	
		No Supplement	+20 μg/mL Methionine
**38**	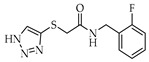	3.1	n.d
**39**	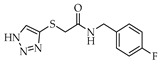	6.3	50
**40**	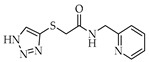	12.5	>50
**41**	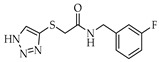	6.3	n.d
**42**	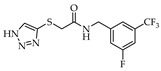	50	n.d
**43**	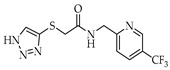	25	n.d
**44**	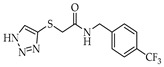	50	n.d
**45**	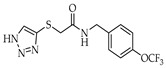	100	n.d
**46**	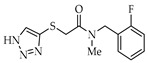	50	n.d

n.d = not determined.

**Table 3:** MIC (μg/mL, left) and accumulation (right) of analogs **50**–**59** against *E. coli* K12.

Compound	Structure	*E. coli* K12 MIC_90_ (μg/mL)	Whole Cell Accumulation in *E. coli* (μM/10^10^ CFU) ^a^
**50**	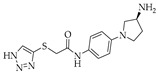	>200	9.0 ± 2
**53**	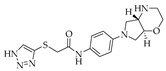	>200	19 ± 1
**56**	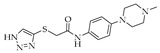	>200	4.7 ± 2
**58**	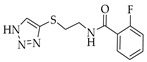	12.5	7.0 ± 1
**59**	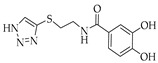	50	1.0 ± 0.1

^a^ = Accumulation values of Ciprofloxacin = 75 ± 4 μM/10^10^ CFU and compound **1** = 20 ± 2 μM/10^10^ CFU.

**Table 4:** MIC (μg/mL) of synthesized analogs **62**–**71** against *E. coli* K12.

Compound	Structure	*E. coli* K12 MIC_90_ (μg/mL)
**62**	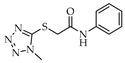	>200
**63**	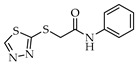	>200
**64**	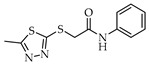	>200
**65**	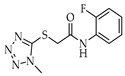	>200
**66**	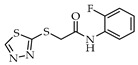	>200
**67**	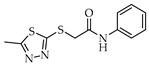	>200
**68**	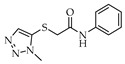	>200
**69**	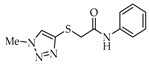	>200
**70**	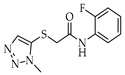	>200
**71**	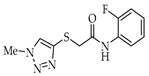	>200

**Table 5:** Solubility and ADME properties of lead TATs.

Compounds	Avg. Sol *^a^* (µg/mL)	Plasma Stability (Mouse) t1/2 (h)	Plasma Stability (Human) t1/2 (h)	Metabolic Stability (Mouse)		Metabolic Stability (Human)	
				t1/2 (h)	Cl_int_ (mL/Min/Kg)	t1/2 (h)	Cl_int_ (mL/min/Kg)
**Verapamil**	39 ± 1.0	2.0 ± 0.1	0.4 ± 0.0	1.1 ± 0.1	51.1	1.5 ± 0.1	14.0
**1**	16 ± 0.8	0.39 ± 0.01	34.8 ± 0.3	0.27 ± 0.01	208.8	3.0 ± 0.2	7.0
**2**	18 ± 2.3	2.5 ± 0.2	36.3 ± 5.5	0.65 ± 0.03	87.4	4.7 ± 0.4	4.4
**3**	19 ± 1.5	1.10 ± 0.02	23.0 ± 1.1	1.13 ± 0.05	50.5	3.7 ± 0.3	5.7
**9**	20 ± 0.3	2.7 ± 0.2	19.9 ± 1.9	0.71 ± 0.02	80.4	2.7 ± 0.2	7.7
**25**	21 ± 1.8	13.3 ± 1.6	32.1 ± 5.4	2.0 ± 0.1	29.1	3.6 ± 0.3	5.8

^*a*^ = Solubility performed at pH = 7.4. Data are presented as average of triplicate replicates ± standard deviation.

## Data Availability

All the data generated in the current research work has been included in the manuscript.

## Supplementary Materials

The following are available online. Analytical data are given for synthesized compounds, Table S1. Solubility of Lead compounds **1**, **2**, **3**, **9** and **25**, Table S2. In vivo pharmacokinetics profile of compound **25**, Table S3. LC-MS/MS methods for whole cell accumulation, Figure S1. LogD vs. TPSA of TATs. References [30,31] are cited in the supplementary materials.
