# Supplementary material for: Synthesis and Structure–Activity Relationship of Thioacetamide-Triazoles against Escherichia coli
Source: Molecules. 2022 Feb 24;27(5):1518. doi: 10.3390/molecules27051518 (PMC8911640; doi:10.3390/molecules27051518)
Supplement: Supplementary file 1 [file molecules-27-01518-s001.zip › molecules-1579361-supplementary.pdf]

Supporting information

for

Synthesis and structure-activity relationship of thioacetamide-triazoles (TATs) against *Escherichia coli*

Suresh Dharuman, Miranda J. Wallace, Stephanie M. Reeve, Jürgen B. Bulitta, and Richard E. Lee*

Table of Content: **Page No.**

Compound synthesis and analytical data S2-10

Supplemental Table S1 S11

Supplemental Table S2 S12

Supplemental Table S3 S12

Supplemental Figure S1 S13

References S14

General procedure A: To a stirred solution of substituted amine (0.90 mmoL) in CH_2Cl_2 (2.0 mL) was added triethylamine (1.35 mmoL) and chloroacetyl chloride (1.08 mmoL) at 0 °C. After stirring the reaction mixture for 1h, the reaction mixture is diluted with CH_2Cl_2 (10 mL), washed with $NaHCO_3$, the organic layer was dried over Na_2SO_4, and then concentrated under high vacuum to give crude chloroacetyl derivative. To a crude chloroacetyl derivative in THF (2 mL) was added sodium 1*H*-1,2,3-triazole-5-thiolate (0.054 mmoL), the mixture was heated at 70 °C for 1h. The solids were filtered off and the filtrate was evaporated under high vacuum to give crude product. The crude product was purified over silica-gel column chromatography (Eluents: 20 – 50% EtOAc in hexane) to produce pure compound.

2-[(1*H*-1,2,3-triazol-4-yl)sulfanyl]-*N*-(pyridin-4-yl)acetamide (**10**): Compound **10** (100 mg, 36%) was synthesized by following general procedure A, as a white solid. m.p. 187 – 188; ^{1}H NMR (500 MHz, CD_3OD) δ 8.39 (d, J = 5.6 Hz, 2H), 7.87 (s, 1H), 7.62 (d, J = 5.6 Hz, 2H), 3.75 (s, 2H); ^{13}C NMR (125 MHz, CD_3OD) δ 38.7, 113.7, 131.1, 137.9, 146.5, 149.3, 168.9; HRMS (ESI) m/z calcd for $C_9H_{10}N_5OS$ $[M+H]^+$, 236.0606; found, 236.0609.

2-[(1*H*-1,2,3-triazol-4-yl)sulfanyl]-*N*-(3-cyanopyridin-2-yl)acetamide (**12**): Compound **12** (200 mg, 43%) was synthesized by following general procedure A, as a white solid. m.p. 113 – 114 °C; ^{1}H NMR (500 MHz, CD_3OD) δ 8.64 (dd, J = 4.9, 1.8 Hz, 1H), 8.19 (dd, J = 7.8, 1.8 Hz, 1H), 7.95 (s, 1H), 7.40 (dd, J = 7.8, 4.9 Hz, 1H), 3.84 (s, 2H); ^{13}C NMR (125 MHz, CD_3OD) δ 38.0, 104.7, 115.0, 121.0, 142.5, 152.2, 168.7; HRMS (ESI) m/z calcd for $C_{10}H_9N_6OS$ $[M+H]^+$, 261.0559; found, 261.0559.

2-[(1*H*-1,2,3-triazol-4-yl)sulfanyl]-*N*-[2-(trifluoromethyl)phenyl]acetamide (**21**): Compound **21** (335 mg, 88%) was synthesized by following general procedure A, as a white solid. m.p. 83 – 85 °C; ^{1}H NMR (500 MHz, CD_3OD) δ 7.86 (s, 1H), 7.70 (d, J = 7.9 Hz, 1H), 7.63 (d, J = 4.3 Hz, 2H), 7.42 (dt, J = 8.5, 4.2 Hz, 1H), 3.84 (s, 2H); ^{13}C NMR (125 MHz, CD_3OD) δ 169.2, 134.60 (d, J = 2.0 Hz), 132.6, 128.5, 126.5, 126.0 (t, J = 5.2 Hz), 124.7, 122.5, 37.6; HRMS (ESI) m/z calcd for $C_{11}H_{10}F_3N_4OS$ $[M+H]^+$, 303.0527; found, 303.0527.

2-[(1*H*-1,2,3-triazol-4-yl)sulfanyl]-*N*-[4-(trifluoromethyl)phenyl]acetamide (**22**): Compound **22** (320 mg, 84%) was synthesized by following general procedure A, as a white solid. m.p. 163 – 165 °C; ^{1}H NMR (500 MHz, CD_3OD) δ 7.88 (s, 1H), 7.73 (d, J = 8.4 Hz, 2H), 7.59 (dd, J = 7.8, 2.6 Hz, 2H), 3.75 (s, 2H); ^{13}C NMR (125 MHz, CD_3OD) δ 168.4, 141.7, 125.6 (q, J = 3.8 Hz),

125.3 (d, J = 5.0 Hz), 123.19, 119.39, 38.73; HRMS (ESI) m/z calcd for $C_{11}H_{10}F_3N_4OS$ $[M+H]^+$, 303.0527; found, 303.0529.

2-[(1*H*-1,2,3-triazol-4-yl)sulfanyl]-*N*-(4-fluorophenyl)-*N*-methylacetamide (**23**): Compound **23** (300 mg, 91%) was synthesized by following general procedure A, as a white solid. m.p. 95 – 96 ºC; ^{1}H NMR (500 MHz, CD_3OD) δ 7.80 (s, 1H), 7.28 (dd, J = 8.7, 4.9 Hz, 2H), 7.16 (t, J = 8.5 Hz, 2H), 3.57 (s, 2H), 3.23 (s, 3H); ^{13}C NMR (125 MHz, CD_3OD) δ 169.1, 162.1 (d, J = 246.6 Hz), 139.2 (d, J = 3.6 Hz), 131.1, 129.2 (t, J = 6.7 Hz), 116.4 (d, J = 22.7 Hz), 115.5 (d, J = 23.1 Hz), 36.9 (d, J = 26.4 Hz); HRMS (ESI) m/z calcd for $C_{11}H_{12}FN_4OS$ $[M+H]^+$, 267.0716; found, 267.0712.

2-[(1*H*-1,2,3-triazol-4-yl)sulfanyl]-*N*-ethyl-*N*-(2-fluorophenyl)acetamide (**24**): Compound **24** (110 mg, 42%) was synthesized by following general procedure A, as a white solid. m.p. 82 – 83 ºC; ^{1}H NMR (500 MHz, CD_3OD) δ 7.76 (s, 1H), 7.43 (tdd, J = 7.0, 5.0, 1.9 Hz, 1H), 7.33 – 7.12 (m, 3H), 3.69 (ddt, J = 38.3, 13.7, 6.9 Hz, 2H), 3.57 – 3.43 (m, 2H), 1.06 (t, J = 7.2 Hz, 3H); ^{13}C NMR (125 MHz, CD_3OD) δ 168.8, 158.1 (d, J = 248.2 Hz), 130.6 (d, J = 7.9 Hz), 130.4, 128.6 (d, J = 12.8 Hz), 125.2 (d, J = 4.0 Hz), 116.5 (d, J = 20.4 Hz), 44.1, 36.8, 11.5; HRMS (ESI) m/z calcd for $C_{12}H_{14}FN_4OS$ $[M+H]^+$, 281.0872; found, 281.0867.

2-[(1*H*-1,2,3-triazol-4-yl)sulfanyl]-*N*-(pyrimidin-2-yl)acetamide (**27**): Compound **27** (50 mg, 73%) was synthesized by following general procedure A, as a yellow solid. m.p. 136 – 138 ºC; ^{1}H NMR (500 MHz, DMSO-d_6) δ 10.75 (s, 1H), 8.65 (d, J = 4.7 Hz, 2H), 7.92 (s, 1H), 7.18 (t, J = 4.8 Hz, 1H), 4.01 (s, 2H); ^{13}C NMR (125 MHz, DMSO-d_6) δ 38.5, 117.3, 157.9, 158.9, 167.7; HRMS (ESI) m/z calcd for $C_8H_9N_6OS$ $[M+H]^+$, 237.0559; found, 237.0556

2-[(1*H*-1,2,3-triazol-4-yl)sulfanyl]-*N*-(2-fluorophenyl)propenamide (**28**): Compound **28** (220 mg, 67%) was synthesized by following general procedure A, as a white solid. m.p. 123 – 124 ºC; ^{1}H NMR (500 MHz, DMSO-d_6) δ 9.85 (s, 1H), 7.97 (s, 1H), 7.90 – 7.77 (m, 1H), 7.37 – 7.22 (m, 1H), 7.16 (dq, J = 7.5, 2.7, 2.0 Hz, 3H), 4.13 (q, J = 6.9 Hz, 1H), 1.39 (d, J = 6.9 Hz, 3H); ^{13}C NMR (125 MHz, DMSO-d_6) δ 170.5, 154.1 (d, J = 245.3 Hz), 126.9 – 125.7 (m), 124.8 (d, J = 3.6 Hz), 124.6, 124.4, 116.0 (d, J = 19.2 Hz), 46.0, 18.4; HRMS (ESI) m/z calcd for $C_{11}H_{12}FN_4OS$ $[M+H]^+$, 267.0716; found, 267.0710.

2-[(1*H*-1,2,3-triazol-4-yl)sulfanyl]-*N*-(4-fluorophenyl)propenamide (**29**): Compound **29** (320 mg, 97%) was synthesized by following general procedure A, as a white solid. m.p. 153 – 154 ºC; ^{1}H NMR (500 MHz, CD_3OD) δ 7.87 (s, 1H), 7.45 (d, J = 24.9 Hz, 2H), 7.00 (p, J = 10.2, 9.7 Hz, 2H), 3.88 (t, J = 9.6 Hz, 1H), 1.49 (d, J = 7.5 Hz, 3H); ^{13}C NMR (125 MHz, CD_3OD) δ 171.0, 159.4 (d, J = 242.2 Hz), 134.3 (d, J = 2.9 Hz), 121.6 (d, J = 8.0 Hz), 114.9 (d, J = 22.6 Hz), 46.8, 16.6; HRMS (ESI) m/z calcd for $C_{11}H_{12}FN_4OS$ $[M+H]^+$, 267.0716; found, 267.0711.

2-[(1*H*-1,2,3-triazol-4-yl)sulfanyl]-*N*-cyclohexylacetamide (**31**): Compound **31** (310 mg, 76%) was synthesized by following general procedure A, as a white solid. m.p. 118 – 119 ºC; ^{1}H NMR (500 MHz, CD_3OD) δ 7.61 (s, 1H), 3.36 (tt, J = 10.7, 4.0 Hz, 1H), 3.31 (s, 2H), 1.57 – 1.48 (m, 2H), 1.45 (dq, J = 13.4, 3.9 Hz, 2H), 1.33 (dt, J = 12.9, 3.9 Hz, 1H), 1.13 – 0.97 (m, 2H), 0.96 – 0.83 (m, 3H).^{13}C NMR (125 MHz, CD_3OD) δ 24.6, 25.2, 32.1, 32.2, 37.9, 48.7, 131.3, 138.0, 168.7; HRMS (ESI) m/z calcd for $C_{10}H_{17}N_4OS$ $[M+H]^+$, 241.1123; found, 241.1120.

2-[(1*H*-1,2,3-triazol-4-yl)sulfanyl]-*N*-(*tert*-butyl)acetamide (**32**): Compound **32** (490 mg, 76%) was synthesized by following general procedure A, as a white solid. m.p. 101 – 102 ºC; ^{1}H NMR (500 MHz, CD_3OD) δ 7.83 (s, 1H), 3.48 (s, 2H), 1.27 (d, J = 1.5 Hz, 9H); ^{13}C NMR (125 MHz, CD_3OD) δ 168.8, 138.2, 131.5, 38.4, 27.3; HRMS (ESI) m/z calcd for $C_8H_{15}N_4OS$ $[M+H]^+$, 215.0967; found, 215.0965.

2-[(1*H*-1,2,3-triazol-4-yl)sulfanyl]-*N*-cyclopentylacetamide (**33**): Compound **33** (510 mg, 90%) was synthesized by following general procedure A, as a white solid. m.p. 108 – 109 ºC; ^{1}H NMR (500 MHz, CD_3OD) δ 7.84 (s, 1H), 4.17 – 3.97 (m, 1H), 3.54 (s, 2H), 1.84 (dq, J = 13.1, 7.0 Hz, 2H), 1.73 – 1.59 (m, 2H), 1.54 (qd, J = 8.9, 7.3, 5.0 Hz, 2H), 1.37 (ddd, J = 14.5, 7.7, 3.9 Hz, 2H); ^{13}C NMR (125 MHz, CD_3OD) δ 23.4, 23.4, 32.1, 32.1, 37.8, 51.4, 51.4, 131.3, 138.0, 169.1, 169.2; HRMS (ESI) m/z calcd for $C_9H_{15}N_4OS$ $[M+H]^+$, 227.0967; found, 227.0963.

General procedure B: To a stirred solution of acid **36**[1] (0.63 mmoL), EDC (0.63 mmoL), HOBt (0.63 mmoL) and substituted benzylamine (0.69 mmoL) in THF (2 mL) was added di-isopropylethylamine (0.63 mmoL) at room temperature. The reaction mixture was stirred for 15min before it was evaporated under high vacuum to give crude product. The crude product was purified on reverse phase silica-gel column chromatography (Eluents: 0 – 3% acetonitrile in water).

2-[(1*H*-1,2,3-triazol-5-yl)sulfanyl]-*N*-(pyridin-2-ylmethyl)acetamide (**40**): The compound **40** (41 mg, 26%) was synthesized by following general procedure B, as a colorless oil. ^{1}H NMR (500 MHz, D_2O) δ 8.44 (dd, J = 5.5, 1.6 Hz, 1H), 8.04 (td, J = 7.8, 1.7 Hz, 1H), 7.80 (s, 1H), 7.56 – 7.51 (m, 1H), 7.30 (d, J = 8.0 Hz, 1H), 4.45 (s, 2H), 3.56 (s, 2H); ^{13}C NMR (125 MHz, D_2O) δ 37.9, 42.5, 123.7, 124.5, 130.1, 136.8, 142.8, 144.5, 154.1, 171.9; HRMS (ESI) m/z calcd for $C_{10}H_{12}N_5OS$ $[M+H]^+$, 250.0763; found, 250.0755.

2-[(1*H*-1,2,3-triazol-5-yl)sulfanyl]-*N*-(3-fluorobenzyl)acetamide (**41**): Compound **41** (70 mg, 42%) was synthesized by following general procedure B, as a white solid. m.p. 85 – 86 °C; ^{1}H NMR (500 MHz, CD_3OD) δ 7.79 (s, 1H), 7.39 – 7.27 (m, 1H), 7.09 – 6.84 (m, 3H), 4.37 (s, 2H), 3.65 (s, 2H); ^{13}C NMR (100 MHz, CD_3OD) δ 169.7, 162.8 (d, J = 244.5 Hz), 141.0 (d, J = 7.2 Hz), 129.7 (d, J = 8.3 Hz), 122.6 (d, J = 2.9 Hz), 113.6 (d, J = 22.1 Hz), 113.3 (d, J = 21.3 Hz), 42.3 (d, J = 2.0 Hz), 37.3; HRMS (ESI) m/z calcd for $C_{11}H_{12}FN_4OS$ $[M+H]^+$, 267.0716; found, 267.0709.

2-[(1*H*-1,2,3-triazol-5-yl)sulfanyl]-*N*-[3-fluoro-5-(trifluoromethyl)benzyl]acetamide (**42**): Compound **42** (105 mg, 50%) was synthesized by following general procedure B, as a white solid. m.p. 82 – 84 °C; ^{1}H NMR (400 MHz, CD_3OD) δ 7.77 (s, 1H), 7.39 (s, 1H), 7.32 – 7.29 (m, 1H), 7.23 – 7.21 (m, 1H), 4.43 (s, 2H), 3.66 (s, 2H); ^{13}C NMR (100 MHz, CD_3OD) δ 170.0, 162.5 (d, J = 247.6 Hz), 143.1 (d, J = 7.4 Hz), 132.0 (dd, J = 33.1, 8.3 Hz), 124.6, 121.9 (d, J = 3.3 Hz), 120.9 – 118.8 (m), 117.5 (d, J = 22.2 Hz), 113.2 – 106.8 (m), 42.0 (d, J = 1.8 Hz), 37.2; HRMS (ESI) m/z calcd for $C_{12}H_{11}F_4N_4OS$ $[M+H]^+$, 335.0590; found, 335.0586.

2-[(1*H*-1,2,3-triazol-5-yl)sulfanyl]-*N*-{[6-(trifluoromethyl)pyridin-3-yl]methyl}acetamide (**43**): Compound **43** (83 mg, 43%) was synthesized by following general procedure B, as a colorless oil. ^{1}H NMR (500 MHz, CD_3OD) δ 8.62 (d, J = 2.0 Hz, 1H), 7.96 – 7.84 (m, 1H), 7.82 (s, 1H), 7.77 (d, J = 8.1 Hz, 1H), 4.50 (s, 2H), 3.68 (s, 2H); ^{13}C NMR (125 MHz, CD_3OD) δ 170.15, 149.23, 146.23 (q, J = 34.6 Hz), 137.87 (d, J = 105.5 Hz), 136.85, 120.57, 120.18 (d, J = 2.8 Hz), 40.17, 37.31; HRMS (ESI) m/z calcd for $C_{11}H_{11}F_3N_5OS$ $[M+H]^+$, 318.0636; found, 318.0628.

2-[(1*H*-1,2,3-triazol-5-yl)sulfanyl]-*N*-[4-(trifluoromethyl)benzyl]acetamide (**44**): Compound **44** (89 mg, 45%) was synthesized by following general procedure B, as a white solid. m.p. 133 – 135 °C; ^{1}H NMR (400 MHz, CD_3OD) δ 7.80 (s, 1H), 7.59 (d, J = 8.1 Hz, 2H), 7.36 (d, J = 8.0 Hz, 2H), 4.43 (s, 2H), 3.66 (s, 2H); ^{13}C NMR (100 MHz, CD_3OD) δ 169.8, 142.8 (d, J = 1.5 Hz), 129.7 –

128.2 (m), 127.3, 125.5, 124.8 (d, J = 3.8 Hz), 122.8, 42.3, 37.3; HRMS (ESI) m/z calcd for $C_{12}H_{12}F_3N_4OS$ $[M+H]^+$, 317.0684; found, 317.0684.

2-[(1*H*-1,2,3-triazol-5-yl)sulfanyl]-*N*-[4-(trifluoromethoxy)benzyl]acetamide (**45**): Compound **45** (90 mg, 43%) was synthesized by following general procedure B, as a white solid. m.p. 131 – 133 °C; ^{1}H NMR (400 MHz, CD_3OD) δ 7.78 (s, 1H), 7.36 – 7.23 (m, 2H), 7.25 – 7.12 (m, 2H), 4.37 (s, 2H), 3.63 (s, 2H); ^{13}C NMR (100 MHz, CD_3OD) δ 169.6, 148.0 (d, J = 1.9 Hz), 137.4, 128.5, 120.5, 42.0, 37.3; HRMS (ESI) m/z calcd for $C_{12}H_{12}F_3N_4O_2S$ $[M+H]^+$, 333.0633; found, 333.0630.

2-[(1*H*-1,2,3-triazol-5-yl)sulfanyl]-*N*-(2-fluorobenzyl)-*N*-methylacetamide (**46**): Compound **46** (65 mg, 37%) was synthesized by following general procedure B, as a colorless oil. ^{1}H NMR (400 MHz, CD_3OD, 1:2 ratio of rotamers) δ 7.84 (s, 1H, minor rotamer), 7.79 (s, 2H, major rotamer), 7.45 – 6.99 (m, 12H, both rotamers), 4.68 (s, 2H, minor rotamer), 4.62 (s, 4H, major rotamer), 3.96 (s, 2H, minor rotamer), 3.92 (s, 4H, major rotamer), 3.06 (s, 6H, major rotamer), 2.87 (s, 3H, minor rotamer); ^{13}C NMR (100 MHz, CD_3OD) δ 169.3 (d, J = 2.7 Hz), 160.8 (d, J = 245.0 Hz), 129.9 – 128.9 (m), 128.7 (d, J = 4.0 Hz), 124.1 (dd, J = 26.0, 3.6 Hz), 123.3 (d, J = 14.9 Hz), 122.9 (d, J = 14.3 Hz), 115.1 (d, J = 21.6 Hz), 114.7 (d, J = 21.9 Hz), 44.5 (d, J = 4.2 Hz), 36.2 (d, J = 1.9 Hz), 34.8, 32.7; HRMS (ESI) m/z calcd for $C_{12}H_{14}FN_4OS$ $[M+H]^+$, 281.0872; found, 281.0872.

tert-Butyl (*S*)-[1-(4-nitrophenyl)pyrrolidin-3-yl]carbamate (**49**): To a stirred solution of 4-nitro-fluorobenzene **47** (500 mg. 3.54 mmol) and boc-protected pyrrolidine **48** (990 mg, 5.32 mmoL) in acetonitrile (10 mL) was added *N,N*-diisopropylethylamine (1.23 mL, 7.09 mmol) at room temperature. The reaction mixture was heated at 90 °C for 5h. Evaporated the reaction mixture to give crude product which was purified on silica-gel column chromatography (Eluents: 30% EtOAc in hexane) to yield pure product **49** (180 mg, 16%) as a yellow solid. ^{1}H NMR (500 MHz, CD_3OD) δ 8.12 (d, J = 9.3 Hz, 2H), 6.62 (d, J = 9.3 Hz, 2H), 4.38 – 4.24 (m, 1H), 3.70 (dd, J = 10.6, 6.4 Hz, 1H), 3.57 (dt, J = 10.2, 7.1 Hz, 1H), 3.49 (ddd, J = 10.2, 7.8, 5.9 Hz, 1H), 3.28 (dd, J = 10.6, 4.8 Hz, 1H), 2.37 – 2.25 (m, 1H), 2.04 (dq, J = 13.0, 6.4 Hz, 1H), 1.47 (s, 9H); ^{13}C NMR (125 MHz, CD_3OD) δ 27.3, 30.6, 45.8, 50.2, 53.0, 79.0, 110.3, 119.3, 124.1, 125.7, 136.5, 152.1.

(*S*)-2-[(1*H*-1,2,3-triazol-5-yl)sulfanyl]-*N*-[4-(3-aminopyrrolidin-1-yl)phenyl]acetamide (**50**): A solution of nitro compound **49** (100 mg, 0.36 mmoL) and Raney Ni (500 mg, excess) in THF (2.0 mL) was stirred under H_2 atmosphere for 10h. After the filtration of excess Raney Ni, the reaction mixture was evaporated to give crude amine. To a crude amine (100 mg) in CH_2Cl_2 (2.0 mL) was

added Et_3N (68 µL) and chloroacetyl chloride (38 µL) at 0 ºC and stirred for 0.5 h. Diluted with DCM, washed with $NaHCO_3$ solution (10 mL), organic layer was dried over Na_2SO_4, and concentrated to give crude chloroacetyl derivative. To a crude chloroacetyl derivative (150 mg) in THF (2.0 mL) was added 1,2,3-triazole-5-thionate (78 mg) at room temperature and the reaction mixture was stirred at 70 ºC for 30min. The solids were filtered off and the solution was evaporated to give crude product which was passed through short silica-gel column to obtain 1,2,3-triazole product. A solution of triazole product (60 mg) in 4M HCl in dioxane (1.0 mL) was stirred for 30min at room temperature. Evaporated the solvents and purified on reverse phase silica-gel column chromatography to give pure **50** (22 mg, 21%) as a brown solid. ^{1}H NMR (500 MHz, D_2O) δ 7.96 (s, 1H), 7.15 – 6.93 (m, 2H), 6.72 – 6.52 (m, 2H), 4.02 (tt, J = 6.8, 3.6 Hz, 1H), 3.58 (s, 2H), 3.53 (dd, J = 11.0, 6.1 Hz, 1H), 3.44 (dt, J = 9.6, 7.5 Hz, 1H), 3.36 (dd, J = 11.1, 3.3 Hz, 1H), 3.27 (td, J = 9.3, 4.9 Hz, 1H), 2.40 (ddt, J = 13.8, 8.8, 7.0 Hz, 1H), 2.10 (ddt, J = 12.9, 8.2, 4.5 Hz, 1H); ^{13}C NMR (125 MHz, D_2O) δ 28.9, 39.0, 45.8, 50.4, 51.5, 113.1, 124.2, 126.1, 145.9, 170.0; HRMS (ESI) m/z calcd for $C_{14}H_{19}N_6OS$ $[M+H]^+$, 319.1341; found, 319.1341.

tert-Butyl (4a*S*,7a*S*)-6-(4-nitrophenyl)hexahydropyrrolo[3,4-b][1,4]oxazine-4(4a*H*)-carboxylate (**52**): Compound **52** (30 mg, 21%) was synthesized following similar protocol as used in **49**. ^{1}H NMR (500 MHz, $CDCl_3$) δ 8.07 (d, J = 9.4 Hz, 2H), 6.62 – 6.27 (m, 2H), 4.13 (s, 1H), 4.00 (ddd, J = 11.9, 3.9, 1.5 Hz, 1H), 3.88 – 3.80 (m, 1H), 3.76 – 3.63 (m, 2H), 3.59 (dd, J = 8.8, 7.4 Hz, 1H), 3.52 (t, J = 10.5 Hz, 1H), 3.24 (t, J = 9.2 Hz, 1H), 3.21 – 3.12 (m, 1H), 2.99 (ddd, J = 13.4, 12.0, 3.8 Hz, 1H), 1.45 (s, 9H); ^{13}C NMR (125 MHz, $CDCl_3$) δ 28.4, 44.8, 48.1, 50.1, 58.3, 67.5, 79.1, 81.3, 110.1, 126.3, 137.6, 151.6, 155.7.

2-[(1*H*-1,2,3-triazol-5-yl)sulfanyl]-*N*-{4-[(4a*S*,7a*S*)-hexahydropyrrolo[3,4-b][1,4]oxazin-6(2*H*)-yl]phenyl}acetamide (**53**): Compound **53** (30 mg, 22%) was synthesized from **52** following similar protocol as used in **50**. ^{1}H NMR (500 MHz, D_2O) δ 7.96 (s, 1H), 7.05 (d, J = 8.6 Hz, 2H), 6.54 (d, J = 8.5 Hz, 2H), 4.21 (dd, J = 13.3, 4.0 Hz, 1H), 4.11 (q, J = 9.1 Hz, 1H), 4.01 – 3.91 (m, 1H), 3.71 – 3.61 (m, 2H), 3.57 (s, 2H), 3.56 – 3.42 (m, 2H), 3.38 – 3.26 (m, 2H), 3.20 (t, J = 9.0 Hz, 1H); ^{13}C NMR (125 MHz, D_2O) δ 39.0, 43.9, 45.9, 47.6, 56.4, 64.5, 75.9, 111.7, 124.3, 125.9, 145.2, 170.0; HRMS (ESI) m/z calcd for $C_{16}H_{21}N_6O_2S$ $[M+H]^+$, 361.1447; found, 361.1439.

2-[(1*H*-1,2,3-triazol-5-yl)sulfanyl]-*N*-[4-(4-methylpiperazin-1-yl)phenyl]acetamide (**56**): To a stirred solution of chloraectyl derivative[2] (80 mg, 0.30 mmoL) in THF (3.0 mL) was added sodium-1,2,3-triazole-5-thiolate **6** (44 mg, 0.36 mmoL) at room temperature and heated at 70 ºC for 30min.

Solids were filtered off and the solution was evaporated under high vacuum to give crude product. The crude product was dissolved in Acetone (10 mL) and MeOH (2 mL) to precipitate out pure compound **56** (60 mg, 60%) as a white solid. ^{1}H NMR (500 MHz, CD_3OD) δ 7.85 (s, 1H), 7.52 – 7.28 (m, 2H), 7.12 – 6.70 (m, 2H), 3.69 (s, 2H), 3.24 – 3.07 (m, 4H), 2.77 – 2.55 (m,4H), 2.39 (s, 3H); ^{13}C NMR (125 MHz, CD_3OD) δ 38.7, 44.6, 48.4, 48.8, 54.5, 116.4, 121.2, 130.8, 131.5, 148.2, 167.8; HRMS (ESI) m/z calcd for $C_{15}H_{21}N_6OS$ $[M+H]^+$, 333.1498; found, 333.1491.

2-[(1*H*-1,2,3-triazol-5-yl)sulfanyl]ethan-1-amine (**57**):

To a solution of 1,2,3-triazole-5-thionate (50 mg, 0.406 mmoL) in dry THF (2 mL) was added *tert*-butyl(2-bromoethyl)carbamate (91 mg, 0.406 mmoL) at room temperature and the reaction mixture was heated at 80 °C for 3h. The reaction mixture was evaporated under reduced pressure to give crude product which was purified on silica-gel column chromatography to yield pure boc compound. The boc compound (60 mg) was dissolved in 4M HCl in dioxane (1 mL) and 1M HCl in water (0.1 mL) and stirred at room temperature for 1h. Evaporated the reaction mixture and washed with acetone to give pure compound **57** (30 mg, 58%) as a white solid. ^{1}H NMR (500 MHz, D_2O) δ 7.98 (s, 1H), 3.21 – 2.98 (m, 4H); ^{13}C NMR (125 MHz, D_2O) δ 136.6, 130.3, 38.5, 31.8.

N-{2-[(1*H*-1,2,3-triazol-5-yl)thio]ethyl}-2-fluorobenzamide (**58**):

Compound **58** (23 mg, 24%) was synthesized from **57** following similar protocol as used in **59**. ^{1}H NMR (500 MHz, CD_3OD) δ 7.89 (s, 1H), 7.72 (td, J = 7.6, 1.9 Hz, 1H), 7.55 – 7.50 (m, 1H), 7.31 – 7.17 (m, 2H), 3.60 (t, J = 6.8 Hz, 2H), 3.12 (dd, J = 7.3, 6.4 Hz, 2H); ^{13}C NMR (125 MHz, CD_3OD) δ 165.5, 165.5, 161.0, 159.0, 132.9, 130.1, 124.2, 122.5, 115.9, 39.4, 33.2; HRMS (ESI) m/z calcd for $C_{11}H_{12}FN_4OS$ $[M+H]^+$, 267.0716; found, 267.0713.

N-{2-[(1*H*-1,2,3-triazol-5-yl)sulfanyl]ethyl}-3,4-dihydroxybenzamide (**59**):

To a solution of 3,4-dihydroxybenzoic acid (30.0 mg, 0.195 mmoL), 5-[(2-(chloro-l5-azaneyl)ethyl)sulfanyl]-1*H*-1,2,3-triazole **57** (35.2 mg, 0.195 mmoL), EDC (30.2 mg, 0.195 mmoL), and HOBt (29.8 mg, 0.195 mmoL) in DMF (1.0 mL) was added *N*-ethyl-*N*-isopropylpropan-2-amine (0.1 mL, 0.584 mmoL) at room temperature. The reaction mixture was stirred for 1h. Diluted with 1M HCl soln., the compound was extracted with EtOAc (3 X 10 mL), organic layer was dried over Na2SO4, and concentrated. The crude product was purified on silica-

gel column chromatography to yield **59** (15 mg, 27 %) as a colorless oil. ^{1}H NMR (500 MHz, CD_3CN) δ 7.81 (s, 1H), 7.27 (d, J = 2.2 Hz, 1H), 7.20 (dd, J = 8.4, 2.2 Hz, 1H), 6.84 (d, J = 8.2 Hz, 1H), 3.54 (t, J = 6.7 Hz, 2H), 3.08 (d, J = 6.8 Hz, 2H); ^{13}C NMR (125 MHz, CD_3CN) δ 168.2 148.92, 145.3, 126.8, 40.1, 34.1; HRMS (ESI) m/z calcd for $C_{11}H_{13}N_4O_3S$ [M+H]$^+$, 281.0708; found, 281.0709.

2-[(1-Methyl-1*H*-tetrazol-5-yl)sulfanyl]-*N*-phenylacetamide (**62**): Compound **62** (30 mg, 28%) was synthesized by following general procedure A, as a white solid. ^{1}H NMR (500 MHz, CD_3OD) δ 8.34 – 8.16 (m, 2H), 8.03 (t, J = 7.8 Hz, 2H), 7.81 (t, J = 7.5 Hz, 1H), 4.72 (s, 3H), 3.99 (s, 2H); ^{13}C NMR (125 MHz, CD_3OD) δ 33.7, 38.0, 38.0, 120.4, 124.8, 129.4, 129.4, 139.1, 154.4, 166.3, 166.4; HRMS (ESI) m/z calcd for $C_{10}H_{12}N_5OS$ [M+H]$^+$, 250.0763; found, 250.0757.

2-[(1,3,4-Thiadiazol-2-yl)sulfanyl]-*N*-phenylacetamide (**63**): Compound **63** (68 mg, 92%) was synthesized by following general procedure A, as a white solid. m.p. 109 – 110 ºC; ^{1}H NMR (500 MHz, CD_3OD) δ 10.07 (t, J = 3.5 Hz, 1H), 8.60 – 8.13 (m, 2H), 8.02 (td, J = 7.9, 3.5 Hz, 2H), 7.81 (t, J = 7.3 Hz, 1H), 4.99 (s, 2H); ^{13}C NMR (125 MHz, CD_3OD) δ 38.0, 119.8, 124.2, 128.7, 138.4, 153.8, 165.7, 166.1; HRMS (ESI) m/z calcd for $C_{10}H_{10}N_3OS_2$ [M+H]$^+$, 252.0265; found, 252.0266.

2-[(5-Methyl-1,3,4-thiadiazol-2-yl)sulfanyl]-*N*-phenylacetamide (**64**): Compound **64** (63 mg, 81%) was synthesized by following general procedure A, as a white solid. m.p. 153 – 154 ºC; ^{1}H NMR (500 MHz, DMSO-d_6) δ 10.35 (s, 1H), 7.57 (d, J = 8.0 Hz, 2H), 7.32 (t, J = 7.7 Hz, 2H), 7.08 (d, J = 7.4 Hz, 1H), 4.26 (s, 2H), 2.67 (s, 3H); ^{13}C NMR (125 MHz, DMSO-d_6) δ 15.7, 38.6, 119.6, 124.1, 129.3, 139.2, 164.9, 165.7, 166.2; HRMS (ESI) m/z calcd for $C_{11}H_{12}N_3OS_2$ [M+H]$^+$, 266.0422; found, 266.0416.

N-(2-Fluorophenyl)-2-[(1-methyl-1*H*-tetrazol-5-yl)sulfanyl]acetamide (**65**): Compound **65** (47 mg, 66%) was synthesized by following general procedure A, as a white solid. m.p. 137 – 139 ºC; ^{1}H NMR (500 MHz, DMSO-d_6) δ 10.17 (s, 1H), 7.86 (ddd, J = 10.1, 5.2, 3.0 Hz, 1H), 7.42 – 7.21 (m, 1H), 7.17 (dt, J = 6.7, 3.2 Hz, 2H), 4.35 (s, 2H), 3.99 (s, 3H); ^{13}C NMR (125 MHz, DMSO-d_6) δ 166.1, 154.9, 153.7, 152.9, 126.1 (d, J = 7.2 Hz), 125.6 – 123.1 (m), 116.0 (d, J = 19.2 Hz), 37.7, 34.2; HRMS (ESI) m/z calcd for $C_{10}H_{11}FN_5OS$ [M+H]$^+$, 268.0668; found, 268.0669.

2-[(1,3,4-Thiadiazol-2-yl)sulfanyl]-*N*-(2-fluorophenyl)acetamide (**66**): Compound **66** (60 mg, 84%) was synthesized by following general procedure A, as a white solid. m.p. 123 – 125 ºC; ^{1}H NMR (500 MHz, DMSO-d_6) δ 10.19 (s, 1H), 9.53 (s, 1H), 7.90 (ddd, J = 9.8, 6.4, 3.9 Hz, 1H), 7.49 – 6.73 (m, 2H), 4.40 (s, 2H); ^{13}C NMR (125 MHz, DMSO-d_6) δ 166.3, 165.5, 154.8, 152.9, 127.0 – 125.4 (m), 124.9 (d, J = 3.5 Hz), 124.2, 116.0 (d, J = 19.3 Hz), 38.4; HRMS (ESI) m/z calcd for $C_{10}H_9FN_3OS_2$ [M+H]$^+$, 270.0171; found, 270.0169.

N-(2-Fluorophenyl)-2-[(5-methyl-1,3,4-thiadiazol-2-yl)sulfanyl]acetamide (**67**): Compound **67** (72 mg, 95%) was synthesized by following general procedure A, as a white solid. m.p. 111 – 113 ºC; ^{1}H NMR (500 MHz, CD_3OD) δ 8.15 – 7.78 (m, 1H), 7.41 – 6.80 (m, 3H), 4.25 (s, 2H), 2.72 (s, 3H); ^{13}C NMR (125 MHz, CD_3OD) δ 167.2, 166.9, 165.4, 155.0, 153.0, 125.6 (d, J = 7.7 Hz), 124.0 (d, J = 3.8 Hz), 123.7 (d, J = 1.3 Hz), 115.0 (d, J = 19.6 Hz), 37.2, 13.9; HRMS (ESI) m/z calcd for $C_{11}H_{11}FN_3OS_2$ [M+H]$^+$, 284.0328; found, 284.0330.

N-(2-Fluorophenyl)-2-[(1-methyl-1*H*-1,2,3-triazol-5-yl)sulfanyl]acetamide (**70**) and *N*-(2-fluorophenyl)-2-[(1-methyl-1*H*-1,2,3-triazol-4-yl)sulfanyl]acetamide (**71**):

To a stirred solution of **1** (270 mg, 1.07 mmoL) in acetonitrile (7.0 mL) was added Cs_2CO_3 (384 mg, 1.17 mmoL) and iodomethane (0.080 mL, 1.17 mmoL) and stirred at room temperature for 2h. Quenched with water (5.0 mL), extracted with EtOAc (2 X 20 mL), combined organic layer was dried over Na_2SO_4, and concentrated under high vacuum to give crude product. Chromatographic (eluents: 40% EtOAc in hexane) purification on silica-gel yielded **70** (120 mg, 42%) and **71**(110 mg, 38%).

70: ^{1}H NMR (500 MHz, $CDCl_3$) δ 9.50 – 9.10 (m, 1H), 8.35 (td, J = 8.1, 1.6 Hz, 1H), 7.55 (s, 1H), 7.22 – 6.92 (m, 3H), 4.22 (s, 3H), 3.79 (s, 2H); ^{13}C NMR (125 MHz, $CDCl_3$) δ 166.8, 152.6 (d, J = 243.5 Hz), 140.5, 134.3, 126.3 (d, J = 10.3 Hz), 126.5 – 124.5 (m), 121.6, 114.8 (d, J = 19.1 Hz), 42.0 (d, J = 1.8 Hz), 38.3; HRMS (ESI) m/z calcd for $C_{11}H_{12}FN_4OS$ [M+H]$^+$, 267.0716; found, 267.0713.

71: ^{1}H NMR (500 MHz, $CDCl_3$) δ 9.56 (s, 1H), 8.28 (td, J = 8.1, 1.7 Hz, 1H), 7.58 (s, 1H), 7.21 – 6.90 (m, 3H), 4.12 (s, 3H), 3.81 (s, 2H); ^{13}C NMR (125 MHz, $CDCl_3$) δ 167.2, 152.9 (d, J = 244.7 Hz), 139.5, 126.3 (d, J = 10.5 Hz), 125.5 – 121.8 (m), 115.0 (d, J = 19.0 Hz), 38.7, 37.2; HRMS (ESI) m/z calcd for $C_{11}H_{12}FN_4OS$ [M+H]$^+$, 267.0716; found, 267.0710.

Table S1. Solubility of Lead compounds **1**, **2**, **3**, **9**, and **25**.

	Solubility[3]							
**Compounds**	**pH**	**Avg. Sol (µg/ml)**	**SD Sol**	**MW**	**LOD (mM)**	**Avg. Sol (µM)**	**SD Sol (µM)**	**%Soluble (%)**
**Albendazole**	7.4	5.33	0.22	265.3	100	20.1	0.8	20.1
**Carbamazepine**	7.4	20.12	0.79	236.3	100	85.1	3.4	85.1
**Ranitidine**	7.4	27.24	0.84	350.9	100	77.6	2.4	77.6
**Verapamil**	7.4	39.04	0.96	491.1	100	79.5	2.0	79.5
**1**	7.4	16.45	0.82	252.3	100	65.2	3.2	65.2
**2**	7.4	18.52	2.27	252.3	100	73.4	9.0	73.4
**3**	7.4	19.00	1.46	259.3	100	73.3	5.6	73.3
**9**	7.4	20.57	0.31	235.3	100	87.4	1.3	87.4
**25**	7.4	21.06	1.85	241.3	100	87.3	7.7	87.3

Table S2. *In vivo* pharmacokinetics profile of compound **25**.

Compound	Route	Dose (mg/kg)	C^0 (ng/mL)	AUC_{last} (h*ng/mL)	AUC_{inf} (h*ng/mL)	$T_{(1/2)}$ h	CL (mL/min/kg)	Vss (L/kg)
**25**	IV	10	7712.12	2798.62	2807.22	0.55	59.37	1.16

Table S3. LC-MS/MS methods for whole cell accumulation.

	Cone Voltage (V)	Collision Energy (eV)	Mass A	Mass Transition
Ciprofloxacin	40	20	333	333.149 → 288.152
**1**	20	15	253	253.054 → 142.007
**50**	20	15	319	319.136 → 319.136
**53**	20	20	361	361.142 → 220.147
**56**	20	20	333	333.154 → 190.133
**58**	20	15	267	267.071 → 166.066
**59**	20	15	281	281.072 → 145.056

Figure S1. LogD vs TPSA of TATs.

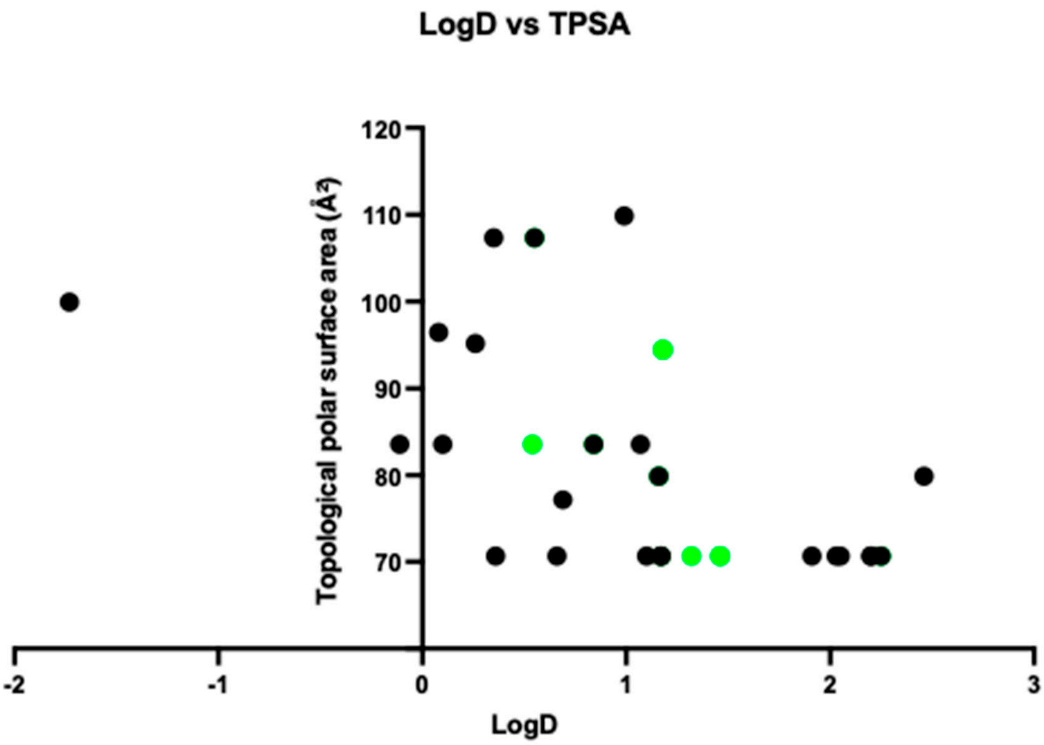

MICs ≤ 3.1 µg/mL are shown in green

References:

1. Drysdale, M. J.; Pritchard, M. C.; Horwell, D. C., Rationally designed "dipeptoid" analogs of CCK. Acid mimics of the potent and selective non-peptide CCK-B receptor antagonist (CI-988). *Journal of Medicinal Chemistry* **1992,** *35* (14), 2573-2581.
2. Liu, H.; Li, Y.; Wang, X. Y.; Wang, B.; He, H. Y.; Liu, J. Y.; Xiang, M. L.; He, J.; Wu, X. H.; Yang, L., Synthesis, preliminary structure-activity relationships, and in vitro biological evaluation of 6-aryl-3-amino-thieno[2,3-b]pyridine derivatives as potential anti-inflammatory agents. *Bioorganic & medicinal chemistry letters* **2013,** *23* (8), 2349-52.
3. Avdeef, A., High-Throughput Measurements of Solubility Profiles. In *Pharmacokinetic Optimization in Drug Research*, 2001; pp 305-325.
